# Genioplasty using a simple CAD/CAM (computer-aided design and computer-aided manufacturing) surgical guide

**DOI:** 10.1186/s40902-015-0044-y

**Published:** 2015-11-24

**Authors:** Se-Ho Lim, Moon-Key Kim, Sang-Hoon Kang

**Affiliations:** Department of Oral and Maxillofacial Surgery, National Health Insurance Service Ilsan Hospital, 100 Ilsan-ro, Ilsan-donggu, Goyang, Gyeonggi-do 410-719 Republic of Korea

**Keywords:** Genioplasty, CAD/CAM, Surgical guide

## Abstract

**Background:**

The present study introduces the design and fabrication of a simple surgical guide with which to perform genioplasty.

**Methods:**

A three-dimensional reconstruction of the patient’s cranio-maxilla region was built, with a dentofacial skeletal model, then derived from CT DICOM data. A surgical simulation was performed on the maxilla and mandible, using three-dimensional cephalometry. We then simulated a full genioplasty, in silico, using the three-dimensional (3D) model of the mandible, according to the final surgical treatment plan. The simulation allowed us to design a surgical guide for genioplasty, which was then computer-rendered and 3D-printed. The manufactured surgical device was ultimately used in an actual genioplasty to guide the osteotomy and to move the cut bone segment to the intended location.

**Results:**

We successfully performed the osteotomy, as planned during a genioplasty, using the computer-aided design and computer-aided manufacturing (CAD/CAM) surgical guide that we initially designed and tested using simulated surgery.

**Conclusions:**

The surgical guide that we developed proved to be a simple and practical tool with which to assist the surgeon in accurately cutting and removing bone segments, during a genioplasty surgery, as preoperatively planned during 3D surgical simulations.

## Background

The simulation of orthognathic surgery using three-dimensional (3D) facial skeletal analyses is increasingly popular [1–3]. The patients’ computed tomography (CT) images can be combined to construct three-dimensional models with which to simulate surgery. These simulations allow us to plan the osteotomy line and predict any movement of the jaw. The surface-data generated is converted into a stereolithography (STL) model, which is computer-rendered and then 3D-printed. These processes follow well-established, computer-aided design and computer-aided manufacturing (CAD/CAM) protocols used for medical devices. The result is a 3D-printed orthognathic surgical guide. The fusion of 3D imaging and ever more powerful CAD/CAM technologies is a powerful new driver in allowing surgeons to design, and create, multiple new surgical devices for use in orthognathic surgeries [1, 4, 5].

Surgical simulations provide the opportunity to derive a more accurate preoperative diagnosis and then plan and simulate surgery. Success at this stage is more likely to lead to the esthetic and functional outcomes desired [6, 7]. For our purposes, several surgical devices can be used to establish the osteotomy line and measure the movement of the chin bone in a genioplasty [4, 6, 7]. In this study, we assessed one method of genioplasty, in which we performed a 3D surgical simulation of a mandibular genioplasty [8]. By simulating the osteotomy on a 3D model, we predicted the movements of the cut chin bone and used these data to 3D-print a surgical guiding device. This device could then be used in an actual genioplasty to guide the osteotomy and move the cut bone segment to the intended location.

## Methods

### Surgical simulation and the manufacture of a surgical guide for advancement genioplasty

Using facial CT data, with slices of less than 1 mm, we reconstructed three-dimensional surfaces using the Mimics software (version 14.0, Materialise, Leuven, Belgium), using the CT data imported in DICOM format (Digital Imaging and Communications in Medicine). The patient’s cranio-maxilla region was reconstructed as a three-dimensional image, and then a model of the dentofacial skeleton was generated using the CT DICOM data. We then performed a surgical simulation on the maxilla and the mandible, using three-dimensional cephalometry; at this point, the final surgical treatment plan was established. A genioplasty simulation was then conducted using the 3D model of the mandible, according to the surgical plan. We could now rehearse, in silico, the osteotomy of the mentum, paying close attention to the positions of the mental nerve, the mental formen, and the margin between the osteotomy line and the anterior teeth root apex. The outcome of this simulation allowed us to make any final adjustments to the position of the osteotomy line.

**Fig. 1:**
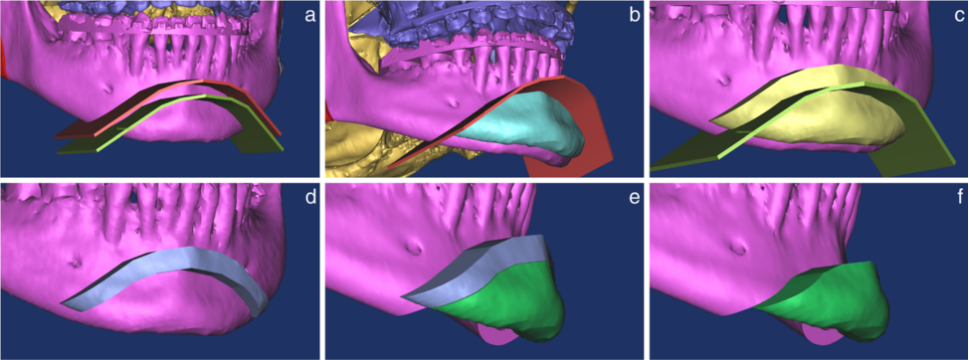
Surgical simulation and the manufacture of a surgical device for advancement genioplasty. **a** The final osteotomy line was established on the chin of the mandible. The copied, initial osteotomy line was moved superiorly, the displacement equaling the thickness of the surgical guide. **b** The cut bone segment was moved anteriorly, according to the desired degree of movement required. **c** The original mandibular data was purged in the displaced segment. The surgical device was designed, after cutting the segment, using the initial osteotomy line. **d** The surgical guide was computer-rendered. **e** Advancement of genioplasty was performed with the designed surgical guide. **f** Simulation surgery was confirmed and then compared with final surgical plan

Based on the surgical simulation results, we designed a surgical guide using the Mimics software. First, we drew the initial osteotomy line and moved the copy of initial osteotomy line, superior to its initial position, to accommodate the guide (Fig. 1a); the guide must be approximately 4–6 mm wide to ensure stability. Next, the modeled mandibular mentum was cut at the line indicated by the superiorly displaced osteotomy line. The cut chin segments were then moved anteriorly, according to the amount of movement desired (Fig. 1b). In advancement genioplasty, the distance of this move is set as the anterior-posterior thickness of the surgical guide. Then, the original mandibular data was erased from the cut chin segment using a Boolean function. The dimensions of the surgical device were initially designed after applying the inferior cut to the chin segment, as per the initial osteotomy line (Fig. 1c); the resultant device “segment” was then modeled, having completed the osteotomy virtually (Fig. 1d). Handily, the anteroposterior thickness of the device will ultimately guide anterior movement, and the osteotomy line is set at the inferior border of the device.

**Fig. 2:**
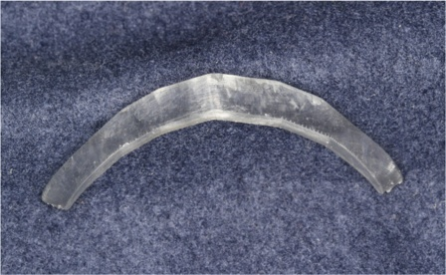
CAD surgical guide for genioplasty, manufactured using a 3D printer

A full simulated advancement of genioplasty surgery was then conducted, using the model of the surgical guide (Fig. 1e, f). The 3D surface model of the surgical device was used to create an STL dataset, which was then rendered and manufactured using a 3D printer (Fig. 2). A biocompatible material was used to manufacture the surgical device, as is mandatory for all such devices with an intended medical use. In the present example, the ZPrinter 350 (3D Systems, Inc., Rock Hill, SC, USA) was used, in conjunction with medical materials [8].

#### Advancement genioplasty using a surgical guide

Having completed the surgery simulations, we progressed to actual surgery. The manufactured device was positioned and fitted to the exposed chin bone during surgery. The “fitting process” is guided by the surgical plan, generated in computer simulations of this phase of the surgery. As mentioned, the anteroposterior width of the surgical guide equaled the desired forward movement of the mandible. Then, mini screws, approximately 2–4 mm longer than the width of the device, were used to fix the device into position; screw positioning depended on the method chosen to stabilize the cut chin bone. For central plate positioning, flanking screws must be used, as in the present example. If the metal plate is to be fixed to the left or right, then only one screw must be used, at the center of the surgical guide. In these cases, the surgeon must pay close attention to ensure that the guide does not slip and rotate, while being attached to the chin bone.

**Fig. 3:**
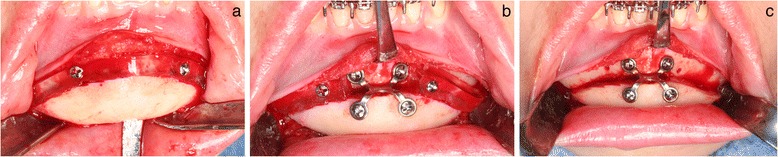
Advancement genioplasty using a surgical guide. **a** Intraoperative view with the surgical guide in position, with two screws for fixation, avoiding the eventual position of the metal plate. The osteotomy line was drawn along the fixed guide. The cut chin bone was displaced anteriorly, according to the anteroposterior width of the surgical device. **b** Using a bur or cutter, a portion of the guide was cut away so that the metal plate could fix the bony segments together. **c** After fixing the plate to the chin, we confirmed its stability and removed the device

Following confirmation of correct fitting, the osteotomy line was drawn, following the inferior border of the guide to check that the surgical device had not moved during the actual surgery. The mentum of the mandible was then cut using a surgical saw against the inferior surface of the surgical device. When cutting, the depth of the saw was adjusted as per the widths of the surgical device and the bone, as measured during the surgical simulation. In this way, we could cut the chin bone cleanly, without lingual soft tissue damage. The cut chin bone was then displaced to the anterior, as per the anteroposterior width of the surgical device (Fig. 3a). In the present example, the chin bone was cut and moved 6 mm. We then confirmed that the chin bone segment had been moved the correct distance before cutting away the central portion of the device where the metal plate was to be fixed (using a bur or cutter (Fig. 3b)). The chin was then fixed to the plate and its stability confirmed before removing the device (Fig. 3c).

### Surgical simulation and manufacturing a surgical guide for reduction genioplasty

**Fig. 4:**
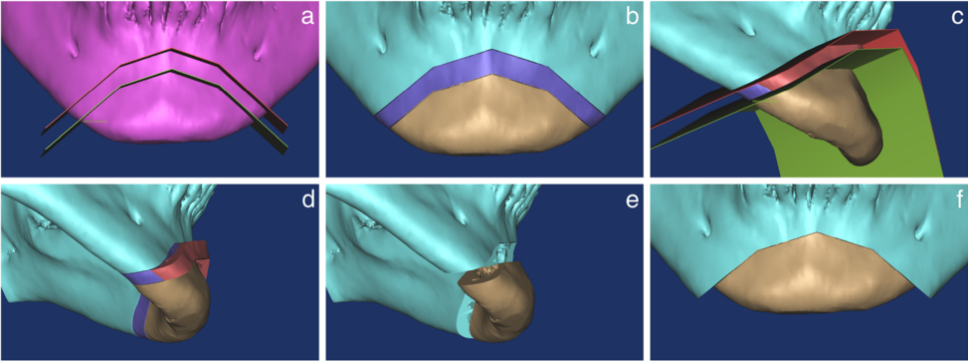
Surgical simulation and the manufacture of a surgical device for reduction genioplasty. **a** The two osteotomy lines were established according to the reduction of chin area required. **b** The gap between the inferior and superior osteotomy lines equaled the thickness of the surgical guide. The distance between the two osteotomy lines dictates the amount of movement required. **c** The original mandibular data was purged in the displaced segment. The surgical device was designed after cutting the segment using the two osteotomy lines. **d** The surgical guide was then computer-rendered. **e** Reduction of genioplasty was performed in silico, using the surgical guide. **f** Simulation surgery was confirmed and compared with final surgical plan

The preparation for reduction genioplasty, prior to simulation, was identical to that described for advancement genioplasty. The two osteotomy lines were determined, with line adjustment depending on the desired reduction of the chin region (Fig. 4a). The mandibular mentum was then cut virtually using the two osteotomy lines. The osteotomy was confirmed and compared with the final surgical plan by imaging segmentation (Fig. 4b). The modeled mandibular mentum was then cut at the superior osteotomy line, and the cut segments moved anteriorly, according to the thickness of the device. Again, the surgical guide should have an anterior-posterior length of about 4–6 mm to ensure stability. The original mandibular data set was then deleted, allowing the surgical device to be designed using the initial two osteotomy lines (Fig. 4c). The computer-aided design of the device (Fig. 4d) was then tested in a simulated surgery for reduction of genioplasty (Fig. 4e, f). Following its successful performance in silico, the model of the device was converted to STL format, extracted, and manufactured using a 3D printer [8].

#### Reduction genioplasty using a surgical guide

**Fig. 5:**
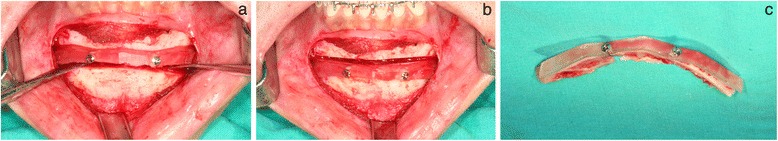
Reduction genioplasty using a surgical guide. **a** Intraoperative view, with the surgical guide in position, fixed by two screws that will ultimately flank the metal plate. The osteotomy line was drawn along the inferior and superior sides of the fixed guide. The mentum of the mandible was cut with a surgical saw, along the inferior side of the surgical device. **b** The chin bone was cut along the superior side of the device. **c** The device was removed, together with the cut chin bone

For this surgery, the superior edge of the surgical device was set as the upper osteotomy line for the chin bone, and the height of the device equaled the intended reduction of the chin bone. Again, the preparation for the reduction genioplasty operation was identical to that for advancement genioplasty. The mentum of the mandible was cut with a surgical saw, along the inferior border of the surgical device (Fig. 5a). The depth of the saw was adjusted as per the widths of the surgical device and bone, measured during the surgical simulation, so that a clean cut of the chin bone was achieved, without lingual soft tissue damage. The chin bone was then cut along the superior border of the device (Fig. 5b), and both the cut chin bone and device were removed. In the present example, 6 mm of the chin bone segment was cut and removed (Fig. 5c). The superiorly moved chin bone segment was then fixed using a metal plate.

## Results and discussion

**Fig. 6:**
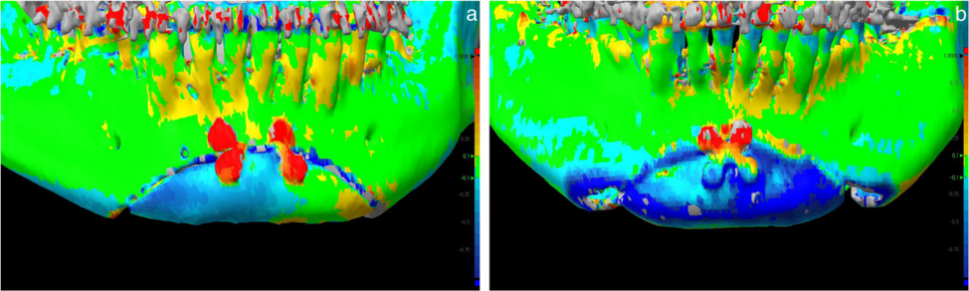
A high level of accuracy achieved between simulated 3D mandible models and postoperative CT images. We superposed models derived for the mandible during the simulated surgery plan, with immediate CT images collected as the outcome of the actual genioplasty. Their superposition, with a color-coded discrepancy map, is shown for **a** advancement genioplasty and **b** reduction genioplasty

Using the method that we described, we successfully performed the osteotomy procedures as planned, during an advancement, and reduction genioplasty, for an orthognathic surgery (Fig. 6). There are several factors to consider when designing a cutting guide for a surgery. If the surgical guide is too thin, the device may break during the surgery, causing safety issues. On the other hand, if the surgical guide is too thick, it may interfere with cutting because it obstructs the position of the surgical saw blade. In addition, the guide could, itself, be cut during the osteotomy, leaving traces of the device material at the surgical site. In the present example, the surgical device was manufactured with a width equal to the desired anterior or superior movement of the chin bone, which made it easy to manufacture and to use during actual surgery.

Recently, there have been several reports of cutting guides used in genioplasty of the mandible [4, 6, 7]. These guides confer several advantages. First, the form and position of the osteotomy line can be adjusted in simulated surgery, which allows the osteotomy line to be preoperatively planned, minimizing the possibility of dental or neural damage. Moreover, using a cutting guide allows the surgeon to adjust the depth when cutting, leading to a non-invasive and stable osteotomy. Cutting guides also make orthognathic surgeries easier by allowing the bone segments to be moved to the planned positions, according to the design of the guide. In a recent multicenter study, the root-mean-square deviation (RMSD) was used to measure the results of orthognathic surgeries using computer-aided surgical simulations (CASS) [9]. Significantly fewer errors were found for genioplasty when surgical guides were used, which led to more favorable surgical outcomes; the largest positional RMSD was 1.0 mm, and the largest orientation RMSD was 2.2°. When a surgical guide was not used, the outcomes varied [9].

There are also drawbacks to using a cutting guide. These include the extra cost of the design and manufacture of a surgical guide. However, these disadvantages diminish as the technologies in this field improve. Given that CAD/CAM-based orthognathic guides are already in use for the maxilla, additional guide models for use in genioplasty can be designed and manufactured more rapidly and therefore more cheaply. We envisage the use of orthognathic surgical guides to cut, and remove, the jaw. In addition, they could be used in conjunction with jaw positioning guides, to allow the surgeon to determine the amount of jaw displacement, using landmark data. More complex guides could even be designed to both hold maxillary and mandibular bone segments in place and link with cutting guides.

Practically, then, it may be more helpful to perform a simulation surgery and then manufacture guides using a 3D printer. Polley et al. reported various CAD/CAM-based surgical guides for use in orthognathic surgeries [4]. Among these devices, those used in genioplasty required more accurate mandibular dental data, and were more complex to design, compared to the method that we now describe [4, 8]. The usefulness of STL guides may also depend on the surgeons’ expertise, and the type of genioplasty, such as zigzag genioplasty [10–12].

Currently, preoperative surgical simulations are widely used and confer an array of advantages [13]. The benefits of faster and more accurate operations when using STL-derived surgical guides are clear [6, 9, 14]. However, there is a cost associated with the time, effort, and materials needed to design and manufacture these devices. Surgical guides may be of most use in complex genioplasty, when it is difficult to accurately reproduce the results of a surgical simulation in the actual operation room with small error; in these scenarios, a surgical guide can be hugely beneficial in guiding the surgeon. We believe that this study will be followed by more thorough research on various designs of surgical guides for use in genioplasty. We expect further research on the development and assessment of orthognathic surgical devices, together with iterative improvements in computing power and software that will expand the possibilities for computer-assisted surgeries. Enhanced navigation and precision robotic surgeries will further increase the power of the now well-established CAD/CAM field in orthognathic surgery [15, 16].

## Conclusions

Using the surgical guide that we have designed, the surgeon can locate the exact point of the chin bone to be cut and can easily perform the osteotomy and genioplasty, according to the simulation plans. Furthermore, the guide can be used as a wafer, fixing the chin bone and clearing the operating view for surgeons. An additional benefit is that this particular surgical guide is small and easy to design, which reduces the time and cost needed to manufacture the device with a 3D printer.

## References

[1] Mazzoni S, Bianchi A, Schiariti G, Badiali G, Marchetti C (2015). Computer-aided design and computer-aided manufacturing cutting guides and customized titanium plates are useful in upper maxilla waferless repositioning. J Oral Maxillofac Surg.

[2] Stokbro K, Aagaard E, Torkov P, Bell RB, Thygesen T (2015). Surgical accuracy of three-dimensional virtual planning: a pilot study of bimaxillary orthognathic procedures including maxillary segmentation. Int J Oral Maxillofac Surg.

[3] Choi JW, Kim BH, Kim HS, Yu TH, Kim BC, Lee SH (2015). Three-dimensional functional unit analysis of hemifacial microsomia mandible—a preliminary report. Maxillofac Plast Reconstr Surg.

[4] Polley JW, Figueroa AA (2013). Orthognathic positioning system: intraoperative system to transfer virtual surgical plan to operating field during orthognathic surgery. J Oral Maxillofac Surg.

[5] Salvato G, Chiavenna C, Meazzini MC (2014). Guide surgery osteotomy system (GSOS) a new device for treatment in orthognathic surgery. J Craniomaxillofac Surg.

[6] Olszewski R, Tranduy K, Reychler H (2010). Innovative procedure for computer-assisted genioplasty: three-dimensional cephalometry, rapid-prototyping model and surgical splint. Int J Oral Maxillofac Surg.

[7] Jegal JJ, Kang SJ, Kim JW, Sun H (2013). The utility of a three-dimensional approach with T-shaped osteotomy in osseous genioplasty. Arch Plast Surg.

[8] Kang SH, Lee JW, Lim SH, Kim YH, Kim MK (2014). Validation of mandibular genioplasty using a stereolithographic surgical guide: in vitro comparison with a manual measurement method based on preoperative surgical simulation. J Oral Maxillofac Surg.

[9] Hsu SS, Gateno J, Bell RB, Hirsch DL, Markiewicz MR, Teichgraeber JF, Zhou X, Xia JJ (2013). Accuracy of a computer-aided surgical simulation protocol for orthognathic surgery: a prospective multicenter study. J Oral Maxillofac Surg.

[10] Keyhan SO, Khiabani K, Hemmat S, Varedi P (2013). Zigzag genioplasty: a new technique for 3-dimensional reduction genioplasty. Br J Oral Maxillofac Surg.

[11] Lee S, Kim BK, Baek RM, Han J (2013). Narrowing and lengthening genioplasty with pedicled bone graft in contouring of the short and wide lower face. Aesthetic Plast Surg.

[12] Sati S, Havlik RJ (2011). An evidence-based approach to genioplasty. Plast Reconstr Surg.

[13] Ahmad Akhoundi MS, Shirani G, Arshad M, Heidar H, Sodagar A (2012). Comparison of an imaging software and manual prediction of soft tissue changes after orthognathic surgery. J Dent (Tehran).

[14] Hierl T, Arnold S, Kruber D, Schulze FP, Humpfner-Hierl H (2013). CAD-CAM-assisted esthetic facial surgery. J Oral Maxillofac Surg.

[15] Li B, Zhang L, Sun H, Shen SG, Wang X (2014). A new method of surgical navigation for orthognathic surgery: optical tracking guided free-hand repositioning of the maxillomandibular complex. J Craniofac Surg.

[16] Choi JW, Jung SY, Kim HJ, Lee SH (2015). Positional symmetry of porion and external auditory meatus in facial asymmetry. Maxillofac Plast Reconstr Surg.

